# Prediction of Individual Melodic Contour Processing in Sensory Association Cortices From Resting State Functional Connectivity

**DOI:** 10.1002/hbm.70409

**Published:** 2025-11-26

**Authors:** Christine Ahrends, Massimo Lumaca, Morten L. Kringelbach, Diego Vidaurre, Peter Vuust

**Affiliations:** ^1^ Center for Music in the Brain, Department of Clinical Medicine Aarhus University, Denmark & Royal Academy of Music Aarhus/Aalborg Denmark; ^2^ Oxford University Centre for Integrative Neuroimaging, Nuffield Department of Clinical Neurosciences University of Oxford Oxford UK; ^3^ Department of Psychiatry University of Oxford UK; ^4^ Department of Clinical Medicine, Center for Functionally Integrative Neuroscience Aarhus University Denmark; ^5^ Centre de Recerca Matemàtica Barcelona Spain

## Abstract

Recent studies suggest that it is possible to predict an individual brain's spatial activation pattern in response to a paradigm from their functional connectivity at rest (rsFC). However, it is unclear whether this prediction works across the brain. We here aim to understand whether individual task activation can be best predicted in local regions that are highly specialised to the task at hand or whether there are domain‐independent regions in the brain that carry most information about the individual. To answer this question, we used fMRI data from participants at rest and during an auditory oddball paradigm. We then predicted individual differences in brain responses to melodic deviants from their rsFC both across the whole brain and within the auditory cortices. Predictability was consistently higher in sensory association cortices: In the local (auditory cortex) parcellation, the best predicted area was the right superior temporal gyrus (STG), an auditory association area, while in the global parcellation, the best predicted network was the bilateral visual association cortex. Our results indicate that individual differences can be predicted in paradigm‐relevant areas or general areas with high inter‐individual variability. Predicting individual task activation from rsFC may be of clinical relevance in cases where patients are unable to carry out a certain task, such as, to inform surgical targets.

## Graphical abstract

Functional activation during a task has been suggested to be part of a shared functional architecture between rest and tasks, which is unique to an individual. We here studied how auditory task activation can be predicted in the whole brain and in the auditory cortices from resting‐state functional connectivity.

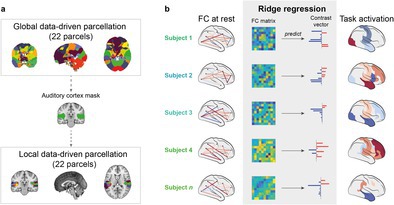

## 1 Introduction

The exact configuration of resting state functional connectivity (rsFC) is unique to an individual, akin to a fingerprint (Biswal et al. 2010; Finn et al. 2015; Horien et al. 2019; Marek et al. 2019). Furthermore, unique patterns that are found in rsFC are also present when an individual is engaged in a task (Finn et al. 2015; Kraus et al. 2021). Individual functional brain networks at rest and during diverse tasks have, in fact, been found to largely overlap, forming a stable functional architecture (Kraus et al. 2021; Krienen et al. 2014; Smith et al. 2009).

Given this stable, unique functional architecture, there have been recent efforts to *predict* what an individual's brain response related to a paradigm will look like based on their rsFC (Cohen et al. 2020; Cole et al. 2016; Langs et al. 2015; Niu et al. 2021; Osher et al. 2019; Parker Jones et al. 2016; Tavor et al. 2016; Tobyne et al. 2018). For instance, using an fMRI dataset of 98 participants and 47 contrasts derived from 7 different paradigms, Tavor et al. (2016) showed that it is possible to predict individual patterns of task activation with great spatial detail and participant specificity. However, a recent evaluation of different methods attempting this prediction has found that most methods fail to perform better than robust baseline models when predicting across the whole cortex (Lacosse et al. 2021). They also found that, among 47 different task contrasts tested, only one could reliably be predicted. Interestingly, Lacosse et al. (2021) also showed that there are differences between brain areas in terms of how well this activation can be predicted. It is unclear why these differences in predictability between brain areas exist.

One reason may be that certain regions are more relevant for the paradigm at hand. Variation in activation within the relevant area may then simply reflect individual differences in the process studied. Another reason may be that some brain networks show more variability across individuals than others, and that this variability is systematic. The functional and structural organisation of these networks would be more sensitive to the influence of idiosyncratic factors (genetics, environment, experience, etc.), and their properties may carry more unique information than others about an individual's activation patterns and behaviour (Lumaca, Baggio, et al. 2021; Lumaca et al. 2019). In biometrics, fingerprints are used because they are thought to be unique to an individual (Pankanti et al. 2002), while other body parts may not be likely to identify a person unequivocally. This may be similar in the brain, where inter‐individual variability may be stronger in some regions than in others.

In the present study, we aim to determine in which parts of the brain rsFC can predict individual differences in melodic processing. People vary in their abilities to perceive and remember melodies (Mullensiefen et al. 2014). This variability has often been linked to differences in musical training, cognitive abilities (mostly working memory and attention), and even personal traits (Correia et al. 2023). However, so far, the role of neural variability in auditory function has been mostly neglected. Are differences in the functional architecture of the auditory cortex linked to (i.e., ‘predict’) differences in melodic processing? To study melodic processing, we employed a paradigm that elicits responses to melodic intervals and melodic contour. Melodic intervals and contour are general features of a musical melody, that is, a sequence of tones. Melodic intervals refer to the size of a pitch change, that is, the step or jump size between two subsequent tones. Melodic contour refers to the direction (up or down) in which it progresses without regard to the exact size of pitch change. The ability to distinguish melodic intervals and contour is a fundamental human ability (Dowling and Fujitani 1971; Peretz and Babaï 1992) that plays a key role in both language and music perception (Patel 2010). Automatic encoding of melodies is already present in humans in the late foetal stage (Granier‐Deferre et al. 2011). Melodic processing varies between individuals based on a number of factors, such as musical expertise (Bailes 2010; Fujioka et al. 2004; Halpern et al. 1998), specific training (Lo et al. 2015) and age (Jeong and Ryu 2016). In the brain, melodic processing is associated with activity in the superior temporal gyrus (STG) (Schindler et al. 2013; Stewart et al. 2006) and there is evidence that this activity is predominantly right‐lateralised (Johnsrude et al. 2000; Lee et al. 2011; Peretz 1990) (though, see Schindler et al. (2013) and Stewart et al. (2008) for work challenging a rightward lateralisation). Since melodic processing is spatially relatively confined to the temporal lobe, this makes it a useful paradigm for studying whether paradigm‐relevant brain areas, that is, the STG or paradigm‐unrelated but highly individual brain areas can be better predicted by rsFC. Additionally, the well‐established background on interindividual differences in melodic processing makes it possible to attribute between‐individual variability, which we aim to predict, to meaningful factors like musical expertise.

To study these differences in predictability between brain areas, we used fMRI recordings where participants first rested and then passively listened to a melodic oddball paradigm. This paradigm used melodic patterns drawn from an artificial musical tuning system, the Bohlen–Pierce (BP) scale (Lumaca and Baggio 2016). Using this dataset, we have previously shown that melodic contour deviants are mainly associated with activity in subregions of the auditory cortices, namely the STG and Heschl's gyri, while interval deviants did not elicit significant activation (Lumaca, Dietz, et al. 2021). This could be explained by the greater difficulty in detecting interval changes relative to contour changes as observed in previous MMN oddball studies with non‐musicians (Schiavetto et al. 1999; Trainor et al. 2002). This difficulty was further compounded by the use of the unfamiliar BP scale, which prevents participants from using their prior knowledge of pitch categories and intervals to predict the next sound in a melodic sequence. As there was no elicited activation, the interval deviant condition was omitted from our analysis. We here created a model to predict each individual's brain response to the melodic contour deviants from their rsFC during the previous scanning session. We hypothesised that inter‐individual differences in functional activation in our paradigm can be better predicted by rsFC in these subregions of the auditory cortices. To determine these differences in predictability of individual activation patterns between the paradigm‐relevant regions and the whole brain, we employed both a whole‐brain parcellation and an auditory cortex sub‐parcellation.

## 2 Methods

### 2.1 Participants

Fifty‐two healthy adult volunteers (33 females, mean age 24.5 years, range 20–34, all right‐handed, English speakers and non‐musicians), with no personal history of neurological and psychiatric disorders and no hearing deficits, consented to the study. Participants were recruited at Aarhus University, leveraging both the university's participant database (http://cfin.sona‐systems.com) and local advertising. The project protocol received ethical approval from De Videnskabsetiske Komitéer for Region Midtjylland, Denmark (sagsnr: 1‐10‐72‐379‐17).

### 2.2 Sample Size

Our sample size is consistent with well‐established research examining relationships between connectivity patterns and behavioural measures, where comparable or even smaller participant groups have been successfully employed. This includes studies by Baldassarre et al. (2012) (*N* = 14), Mattar et al. (2018) (*N* = 22), Tambini et al. (2010) (*N* = 16), Barttfeld et al. (2013) (*N* = 25) and Ventura‐Campos et al. (2013) (*N* = 22). More recent studies have adopted the use of similar cohorts, such as those by Herholz et al. (2016) (*N* = 15), van den Bos et al. (2014) (*N* = 22) and Niu et al. (2021) (*N* = 47). Given this established precedent in the field, we proceeded without conducting a formal power analysis at the study's outset.

### 2.3 Study Design and Data Acquisition

MRI data were acquired on a 3 T MRI scanner (Siemens Skyra). Participants first completed a functional scan at rest (‘resting state data’; 600 volumes for a total acquisition time of 9 min), followed by a structural scan (acquisition time: 10 min duration) and a second functional scan with a paradigm (‘paradigm data’, 1535 volumes for a total acquisition time of 25 min). Positioning the resting‐state scan at the beginning helps minimise fatigue‐related confounds that could compromise the quality of spontaneous BOLD signal fluctuations. Second, the protocol was structured to maximise scanner efficiency, as the functional scan reconstruction process required exactly 10 min to complete—precisely matching the duration of the structural acquisition. During the paradigm scan, participants were presented with an auditory oddball paradigm while watching a subtitled silent documentary. The structural scan consisted of a T1‐weighted high‐resolution imaging sequence using an MP2RAGE sequence (TR = 5000 ms, TE = 2.87 ms and voxel size = 0.9 mm^3^). Functional images were acquired using a fast multi‐band EPI sequence (TR = 1000 ms, TE = 29.6 ms, voxel size = 2.5 mm^3^, interleaved acquisition and acceleration factor 3). The auditory oddball paradigm was presented via MR‐compatible headphones. We presented participants with a stream of melodic patterns: standards (80% frequency), contour deviants (10%) and interval deviants (10%). Each ‘standard’ pattern consisted of five different tones drawn from the equal‐tempered version of the BP scale. In the contour ‘deviants’, the fourth tone violated the surface structure (‘ups’ and ‘downs’) of the sequence, but not the interval size; vice versa for the interval ‘deviants’. To create variation, patterns were randomly transposed at three different registers of the BP scale (low, medium and high) (for more details, see Lumaca, Dietz, et al. 2021; Lumaca et al. 2019).

### 2.4 Pre‐Processing

We used similar, but distinct pre‐processing pipelines for resting state and paradigm data due to the different analysis aims (FC analysis of resting state data and generalised linear model (GLM) analysis of paradigm data).

Resting state data were pre‐processed in FMRIB's Software Library (FSL; Jenkinson et al. 2012). The pipeline consisted of minimal spatial pre‐processing, followed by temporal artefact removal. Namely, skull and neck segments were removed from the images using the BET brain extraction. The first three volumes of all functional scans were discarded. Initial motion correction (realignment) was carried out using MCFLIRT. We set a threshold of 0.5 mm mean relative rms displacement for possible exclusion of participants due to head motion. No participants exceeded this threshold. The functional scans were then high‐pass filtered at 1/2000 Hz and slice‐time corrected for the specific slice acquisition order. We used spatial smoothing with a Gaussian kernel of 6 mm FWHM. We then registered all functional scans to their respective structural scans and to MNI space using FLIRT. For temporal artefact removal, each participant's scanning session (in native space) was decomposed into independent components (ICs) using MELODIC Independent Component Analysis (ICA) (Beckmann 2012). We trained FIX (Griffanti et al. 2014; Salimi‐Khorshidi et al. 2014) to classify ICs into noise and signal based on 10 participants' hand‐labelled session components, which were labelled following guidelines for manual artefact classification (Griffanti et al. 2017). The identified artefactual components, as well as 24 motion regressors, were regressed out of each participant's functional data using unique variance clean‐up. The individual functional scans were then transformed to MNI space to perform ICA on the group level.

Pre‐processing of the paradigm data is described in detail by Lumaca, Dietz, et al. (2021) and Lumaca et al. (2019). Briefly, using SPM12 (r7487), fMRI data were realigned, co‐registered, normalised to MNI space and spatially smoothed (6 mm FWHM). Time series were high‐pass filtered at 1/128 Hz and corrected for serial auto‐correlations using an AR(1) process, including white noise.

### 2.5 Data‐Driven Parcellations

The goal of ICA decomposition on the group level is the creation of a data‐driven parcellation. Group‐level components are voxels that share temporal variance across the time courses of all participants. Each of these group components can be considered a functional parcel. The size and distribution of these components can vary between a small, local cluster of voxels and a distributed network, such as the default mode network (DMN). We therefore refer to the components as parcels rather than regions or networks.

**FIGURE 1:**
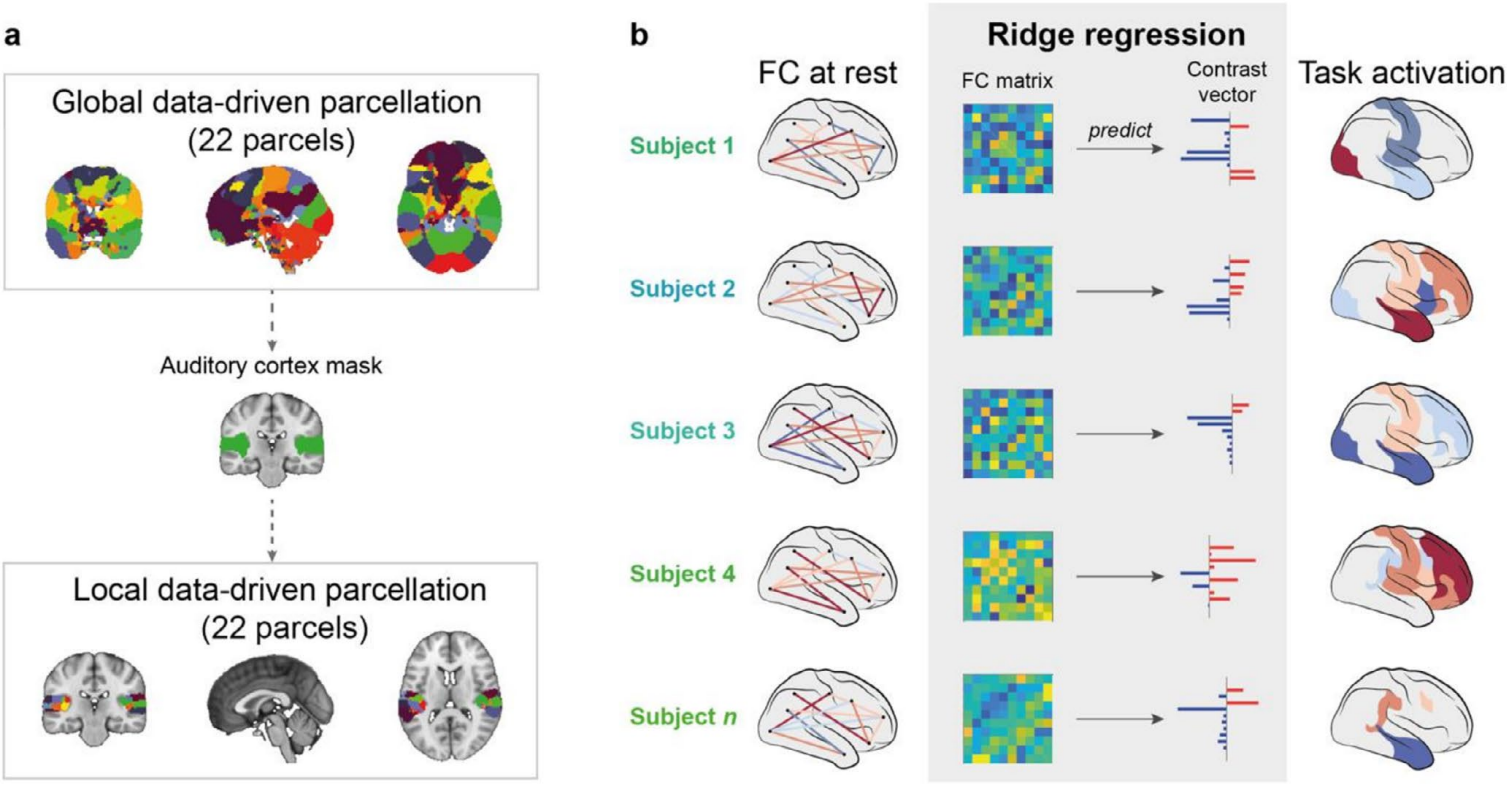
Data‐driven parcellations and regression model. (a) We used two different parcellations to extract time courses of the resting state data and parcellate the paradigm contrast. Both parcellations were created in a data‐driven way by decomposing the fMRI data into 22 independent components. We first created a ‘global’ parcellation using functional data from the whole brain as input (top panel). We then selected one parcel from this parcellation that most closely resembles the bilateral auditory cortices (middle panel). We binarised this parcel to create a mask of the auditory cortex. We then decomposed the functional data again using this mask to limit the spatial input to the auditory cortex. This results in a ‘local’ parcellation of the auditory cortex (bottom panel). Please note that we here show the binarised, non‐overlapping versions of the parcellations for visualisation purposes. These versions are used to parcellate the paradigm contrast. The original versions of each parcellation are weighted and overlapping, and we used these versions to extract time courses from the resting state functional data. (b) We used ridge regression to predict paradigm activation from rsFC. First, all *n* participants' individual rsFC was computed in the two parcellations. The upper triangles of these n FC matrices (each of size *N*_parcels_ × *N*_parcels_) were unwrapped and then used as predictor of the vector (of size 1 × *N*_parcels_) containing each parcel's value in the paradigm contrast.

To create this group ICA parcellation, the voxel‐level resting state data of all participants in MNI space were temporally concatenated. These group time series were then decomposed into ICs using MELODIC ICA (Beckmann et al. 2009). Using this approach, we estimated 25 components across the resting state scanning sessions of all participants. The ICA dimensionality was manually chosen to match typical coarse data‐driven functional parcellations, such as the groupICA25 parcellation from the Human Connectome Project (Smith, Beckmann, et al. 2013). Three of these components were removed as (group‐level) noise components following visual inspection, resulting in 22 parcels for the final parcellation. The resulting whole‐brain (global) parcellation is illustrated in Figure 1a (top panel). Please note that we show a binarised version of the parcellation, in which overlap between parcels has been removed because it is simpler to visualise. In the original parcellation, parcel membership of each voxel across the brain is weighted. We use the original non‐binarised weighted parcellation to extract resting state time courses and the binarised, non‐overlapping version to summarise the paradigm contrast in each region, as explained below.

Since the global parcellation is relatively coarse and might not provide the necessary level of detail to capture individual differences in an auditory oddball paradigm, we created a second, more fine‐grained parcellation of the bilateral auditory cortices. It is well established that the auditory cortices are the most relevant regions for an auditory oddball paradigm such as the one we used here (Opitz et al. 1999, 2002; Schönwiesner et al. 2007; Wible et al. 2001). We refer to this parcellation as ‘local’ parcellation. To be able to compare the two parcellations, we used the same data‐driven approach as for the global parcellation, and we matched the number of ICs (25 parcels for the global parcellation and 25 parcels for the local parcellation). This will segregate the auditory cortex into smaller, functionally specialised regions rather than large‐scale areas or networks as in the global parcellation. It should be noted that there is potentially lower signal‐to‐noise ratio in these small functional clusters compared to the large‐scale networks. Rather than using an anatomically chosen mask of the auditory cortices, we thus chose the component from the global parcellation that had the closest correspondence to the auditory cortices (shown in Figure 1a, centre). We then ran group‐level ICA on all participants' concatenated data only within this auditory cortex mask. This process is summarised in Figure 1a. Similar to the global parcellation, we also decomposed the auditory cortex functional data into 25 components, 3 of which were removed as most closely resembling artefacts. The final auditory cortex sub‐parcellation is shown in Figure 1a (bottom panel). As for the global parcellation, we plot only the binarised, non‐overlapping version of the parcellation as used to parcellate the paradigm contrast. Summary spatial maps of the non‐binarised components for both parcellations can be found in Figures S1 and S2. Our approach of creating a local data‐driven functional parcellation from a global data‐driven functional parcel using ICA is similar to the approach taken by van Oort et al. (2018). The main difference is that in our approach, the time course of the global parcel is not explicitly modelled in the local parcellation, so that local parcels may still contain the time course of the global parcel they are contained within.

### 2.6 rsFC and Paradigm Contrast

To compute rsFC, we first extracted time courses within each parcellation from the resting state scanning session. We used dual regression (Beckmann et al. 2009) to extract participant‐specific time courses of the global and local group‐ICA parcellations. Dual regression consists of a first step, where the group‐level weighted parcels are regressed against each single participant's voxel time courses as spatial regressors using multiple regression to obtain participant‐specific IC time courses; and a second step, in which these participant‐specific time courses are used as temporal regressors to obtain participant‐specific weights of spatial maps of each parcel. The participant‐specific time courses are used to compute rsFC. We used FSLnets (Smith, Vidaurre, et al. 2013) to first standardise the time series and then use Pearson's correlation to compute the pairwise FC between all parcels. The correlations were then *z*‐transformed. This obtained a matrix of size *N*_parcels_ × *N*_parcels_ for each participant, in which each element represents the pairwise rsFC between two parcels. Here, *N*_participants_ (the number of participants) is 52 and *N*_parcels_ (the number of parcels in each parcellation) is 22. We applied the same procedure to both the global and the local parcellation.

The paradigm data were analysed as described by Lumaca, Dietz, et al. (2021) using SPM12. First‐level analysis consisted of a GLM with standard (STD; implicitly modelled) and deviant contour (DC) regressors convolved with a canonical hemodynamic response function (HRF), and realignment parameters to account for head motion. We contrasted the standards with the contour deviants. We then parcellated the contrast map of each individual into the same parcellations as used for the resting state data. Since the paradigm contrast of a participant consists of only one time point, this could not be done using dual regression as for the resting state data. Instead, the parcellations were first binarised and overlap between parcels was removed. We then extracted values within each parcel as the mean of all voxels belonging to that specific parcel. This resulted in a vector of size 1 × *N*_parcels_ for each participant, where the vector elements are summaries of the paradigm contrast value within a given parcel. We extracted values of the paradigm contrast for both the global and the local parcellation.

### 2.7 Predictive Model

To predict individual paradigm activation across the brain, we used a separate ridge regression model for each parcel of the global and local parcellation, respectively. The general idea of the predictive model is illustrated in Figure 1b.

For each model, we used the rsFC of all participants as a predictor. We first reshaped the 3D tensor containing all participants' rsFC matrices of size *N*_participants_ × *N*_parcels_ × *N*_parcels_ by vectorising the upper triangle of each participant's *N*_parcels_ × *N*_parcels_ FC matrix, resulting in a matrix of *N*_participants_ × (*N*_parcels_ × (*N*_parcels_ − 1)/2). We then standardised this matrix column‐wise. The outcome variable of each model is the *N*_participants_ × 1 vector containing individual contrast values within one parcel. We thus fitted *N*_parcels_ (independent) models for each parcellation. In the runs where the outcome variable was paradigm activation in the global parcellation, rsFC in the global parcellation was the predictor, and in the runs where we predicted paradigm activation in the local parcellation, rsFC in the local parcellation was the predictor.

The regression problem is defined as:

$${\hat{y}}_{j}=\beta_{j}X+\varepsilon$$

where *ŷ*_*j*_ is the *N*_participants_ × 1 vector of predicted values in region *j*, *X* are the features from the rsFC matrix, *β*_*j*_ is the regression parameter for parcel *j* and *ε* is the error (residual) term. The parameter *β*_*j*_ is estimated by minimising:

$${\hat{\beta }}_{j}∶=\arg\min_{\beta_{j}}{\left\|y_{j}-\beta_{j}X\right\|}^{2}+\lambda {\left\|\beta_{j}\right\|}^{2}$$

where ${\hat{\beta }}_{j}$ is the new estimate for *β*_*j*_ and *λ* is a regularisation parameter.

We used fourfold nested cross‐validation to optimise the regularisation parameter (inner loop) and evaluate the accuracy on the test set. We performed 100 iterations of this process, where participants were randomly assigned to the different cross‐validation folds. This means that the model found slightly different parameters in each iteration, and the accuracy therefore varies.

We evaluated the model's accuracy in each of these iterations by correlating the model‐predicted values *ŷ*_*j*_ with the actual values *y*_*j*_. This indicates how well the model's prediction of differences between participants in a specific parcel corresponds to the actual variability in this parcel. We used this measure as a basis to identify parcels that can be better predicted than others, as described in detail in the next section.

The predictive model and the following analyses were carried out using MATLAB (MATLAB 2016). Code for ridge regression from FC is available under https://github.com/vidaurre/NetsPredict.

### 2.8 Parcel‐Specific Probabilistic Index

We calculated a parcel‐specific probabilistic index to identify parcels where prediction accuracy was unexpectedly high as compared to all other runs and parcels. Our approach is inspired by the probabilistic indices of abnormality introduced by Marquand et al. (2016). Compared to the approach taken by Marquand et al. (2016), we here aimed to find parcels that are positive outliers in terms of model accuracy with respect to the other parcels and runs, rather than identifying participants and brain regions that can be considered abnormal with respect to a normative population. The probabilistic index is therefore calculated for the parcel‐level correlation coefficients (i.e., the correlation between *ŷ*_*j*_ and *y*_*j*_) instead of the activation values themselves.

The cross‐validation folds used to estimate the model's parameters are randomised 100 times, meaning that we have 100 slightly different iterations of each parcel model. We then calculate the correlation between *ŷ*_*j*_ and *y*_*j*_ for each parcel *j* and each iteration. Next, we fit a normal distribution and the corresponding probability density function (PDF) to all correlation coefficients. Given this function, we can obtain the probability of the correlation coefficient being this particular value or higher by computing the integral of the curve between each obtained correlation coefficient and ∞. We first focus on the right tail of this distribution, containing all values where this probability is smaller than 5% (corresponding to a *z*‐score > 1.96). In other words, we are looking at the top 5% of all predictions of between‐participant variability in all parcels and runs. We then plot in the brain how many of the 100 iterations of each parcel appeared in this right tail of the distribution using Connectome Workbench (Marcus et al. 2011).

Although this measure may be an interesting indicator of prediction accuracy differences between parcels, the random iterations of the model may only coincidentally appear in the tail of this distribution. We therefore next computed a probabilistic index based on the robust best runs of each parcel. We first computed the *z*‐scores of the obtained correlation coefficient for each parcel and each run with respect to all runs and parcels. We then considered the ‘block maxima’ by calculating the 90% trimmed mean of the highest 10% *z*‐scores within each parcel. In other words, we here focused on the (trimmed) right tail of the distribution of each parcel. These block maxima can be described using an extreme value distribution (EVD) and its corresponding PDF. As before, we can calculate the probability of the block max. *z*‐score of a given parcel being the obtained value or higher within the EVD. The resulting probability value given the EVD of block maxima is a robust, network‐specific probabilistic index. This index can be plotted in the brain to identify parcels that are positive outliers in prediction accuracy.

## 3 Results

### 3.1 Within the Auditory Cortex, Individual Task Activation Can Be Best Predicted in Task‐Relevant Right STG

**FIGURE 2:**
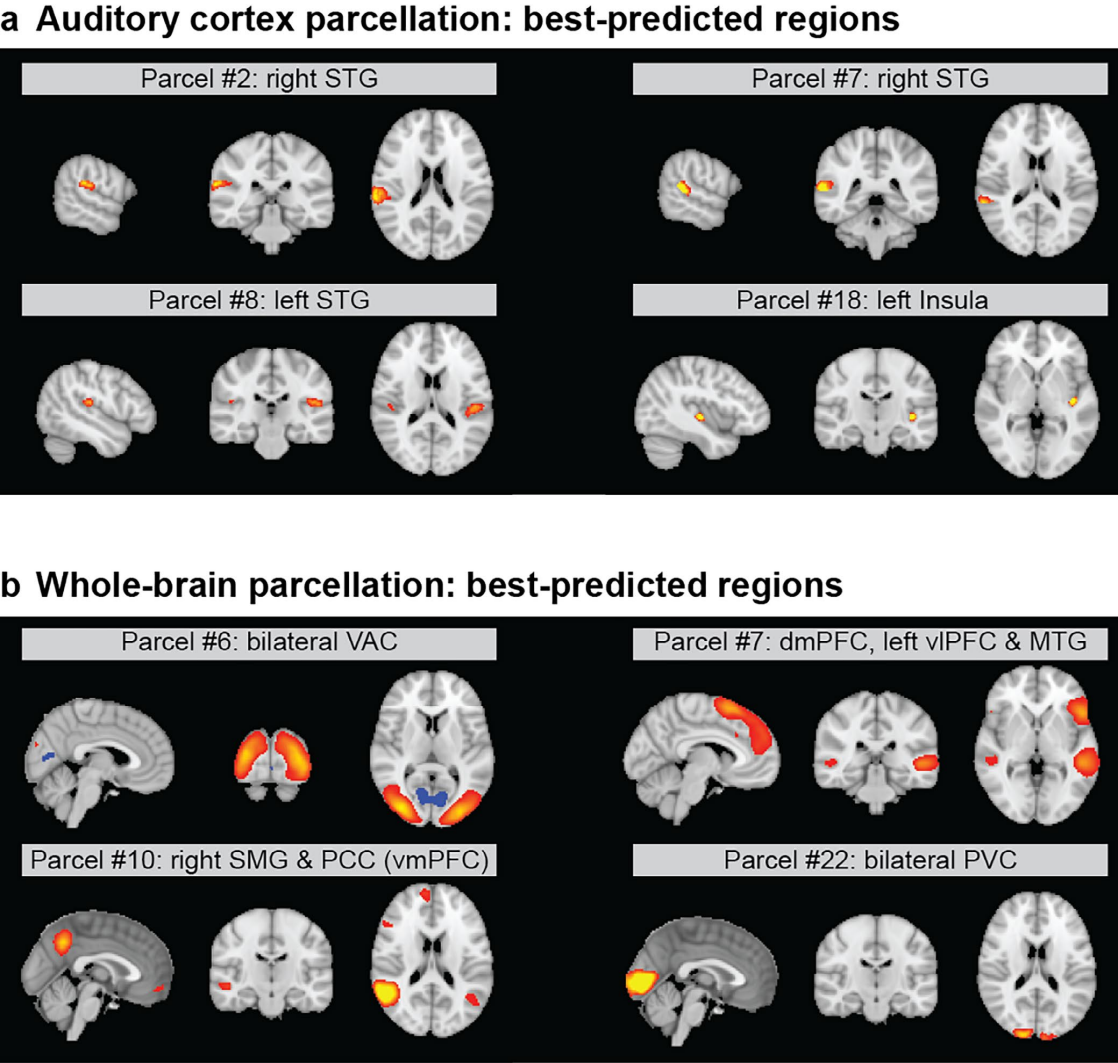
Maps of best predicted regions. The maps show the spatial distribution for the components that were best predicted within each parcellation, that is, the non‐binarised ICA component weights. (a) Best predicted regions within auditory cortex parcellation. (b) Best predicted regions within whole‐brain parcellation. dmPFC, dorsomedial prefrontal cortex; MTG, middle temporal gyrus; PCC, posterior cingulate cortex; PVC, primary visual cortex; SMG, supramarginal gyrus; STG, superior temporal gyrus; VAC, visual association cortex; vlPFC, ventrolateral prefrontal cortex; vmPFC, ventromedial prefrontal cortex.

In the local auditory cortex parcellation, the parcel corresponding to the right STG was the best predicted. The next best predicted regions included the left STG and the posterior part of the left insula. The maps of the best predicted regions within the auditory cortex are shown in Figure 2a.

**FIGURE 3:**
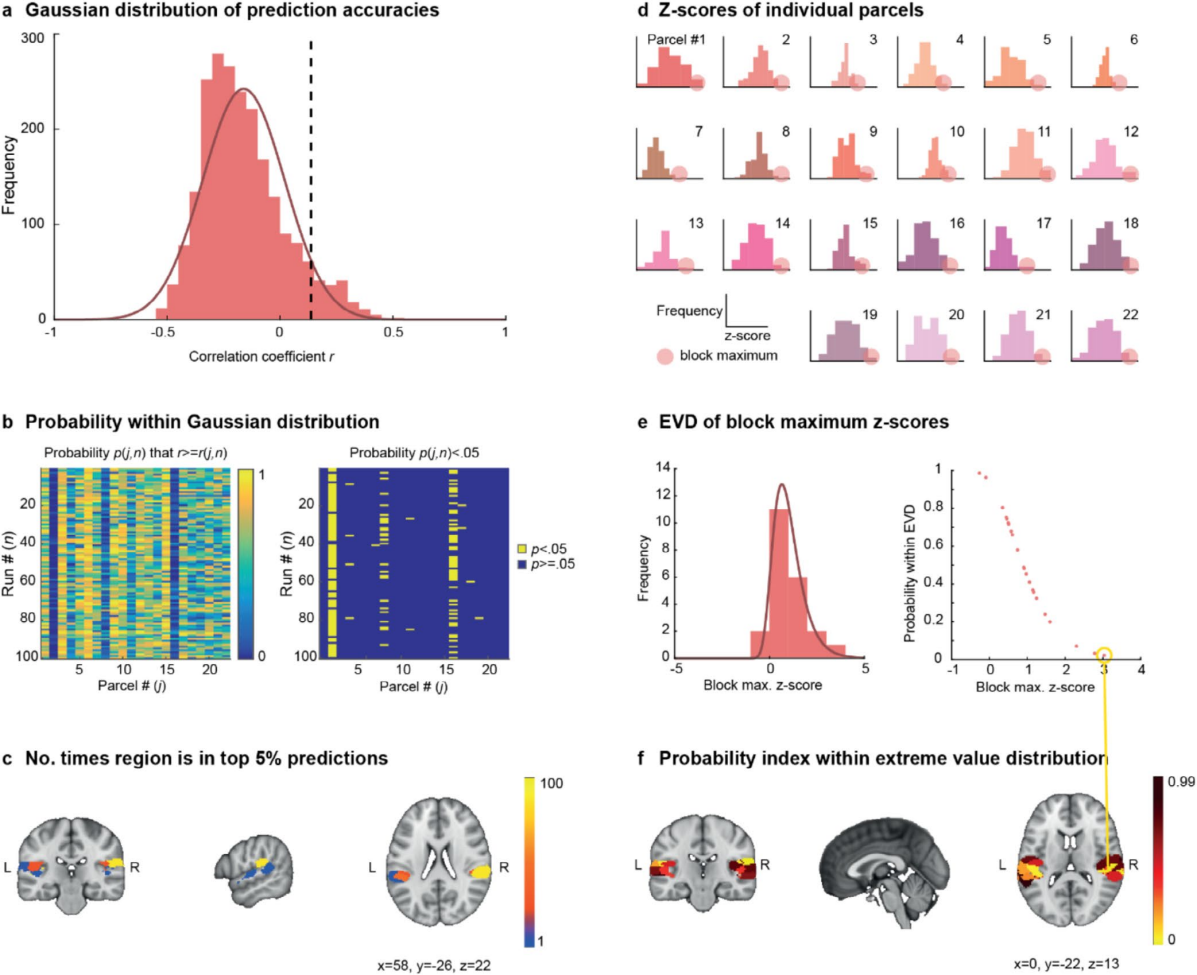
Task activation is robustly better predicted in task‐relevant regions of the auditory cortex. (a) Histogram of prediction accuracies across all 22 parcels and 100 iterations and fitted Gaussian distribution. The dashed line represents the 95th percentile, that is, the top 5% of predictions. (b) Probability of obtaining prediction accuracy by parcel and run. (c) Regions that appear more than once in the top 5% predictions within the auditory cortex. (d) *z*‐scores and block maxima (the 10% best predicted runs) across runs for each parcel. (e) Histogram and fitted extreme value distribution (EVD) for 90% trimmed mean block maximum *z*‐scores from (d). (f) Probability index, that is, probability of obtaining the block max. *z*‐score given the PDF of the EVD from (e) across regions in the auditory cortex parcellation.

We ran each model 100 times, randomly re‐assigning subjects to cross‐validation folds at each iteration. We then considered where each parcel lies in the distribution of prediction accuracies across all iterations and parcels. Figure 3a shows the distribution of all prediction accuracies, as measured by the correlation coefficient between the predicted interindividual variability and the actual interindividual variability. To estimate the probability of obtaining a certain prediction accuracy, we fitted a Gaussian distribution to the observed prediction accuracy values and considered the probability given the PDF of this distribution. Since we were interested in regions where differences between individuals are significantly better predicted than others, we focused on the right tail of this distribution, where the probability of correlation coefficients *r* being equal to or higher than *r(j,n)* for parcel *j* and run *n* is less than 5%. The dashed line represents the 95th percentile, that is, where the probability of obtaining a prediction accuracy greater than or equal to the one measured is 5%, corresponding to a *z*‐score of 1.96. We then looked at these results by parcel and run to understand whether all parcels appear equally often in these top 5% of predictions, or whether a small number of parcels appear consistently over runs. Figure 3b plots these probabilities by parcel and run, indicating that the best predictions are not randomly distributed across parcels but that certain parcels consistently appear in the top 5% of predictions, while others never appear. Projecting these counts for each parcel into the brain (see Figure 3c), we can observe that the region with the highest number of appearances in the top 5% of predictions is the right STG (here Parcel #2), which scores in the 95th percentile in 87 out of 100 runs. Only parcels that appear at least once in this tail of the distribution are highlighted. Heschl's gyrus (Parcels #13 and #14, see also Figure S2), which was previously found to be significantly activated during this task, did not feature in the top predictions (both 0 out of 100 runs).

Since the cross‐validation step introduces randomness in the model and its outcome, single runs of the model are not necessarily a reliable indicator of model performance. To account for this randomness, we next computed a robust, probabilistic index given the best runs within each parcel. Specifically, we computed the 90% trimmed mean of the highest 10% *z*‐scores within each parcel (‘block maximum’, see Figure 3d). We then fitted an EVD to these block maxima (Figure 3e). Given the PDF of this EVD, we can compute a probabilistic index for each IC, indicating the probability within the EVD that the block max. *z*‐score of a given region would be the obtained value or higher. This allows for identifying regions that are positive outliers in terms of prediction accuracy. The relationship between block max. *z*‐scores and the probability within the EVD are shown in Figure 3d,e. In the auditory cortex parcellation, the strongest outlier (i.e., the parcel that is robustly best predicted) is again the right STG (Parcel #2) with a block max. *z*‐score of 3.03 (*p* = 0.02). The probabilistic index of each region in the brain is plotted in Figure 3f. Low values indicate that the highest obtained prediction accuracy is significantly higher than the highest obtained prediction accuracy in other parcels.

### 3.2 Across the Brain, Visual Rather Than Auditory Cortex Activation Can Be Best Predicted

In the global parcellation, we found the bilateral visual association cortices to be the best predicted. The next best predicted parcels contain the bilateral primary visual cortices, the right primary motor cortex, the posterior cingulate cortex, the bilateral hippocampus, the bilateral caudate nuclei, the medial prefrontal cortex and the pars triangularis of the right inferior frontal gyrus. The spatial distributions of each of the best predicted parcels from the global parcellation are shown in Figure 2b.

**FIGURE 4:**
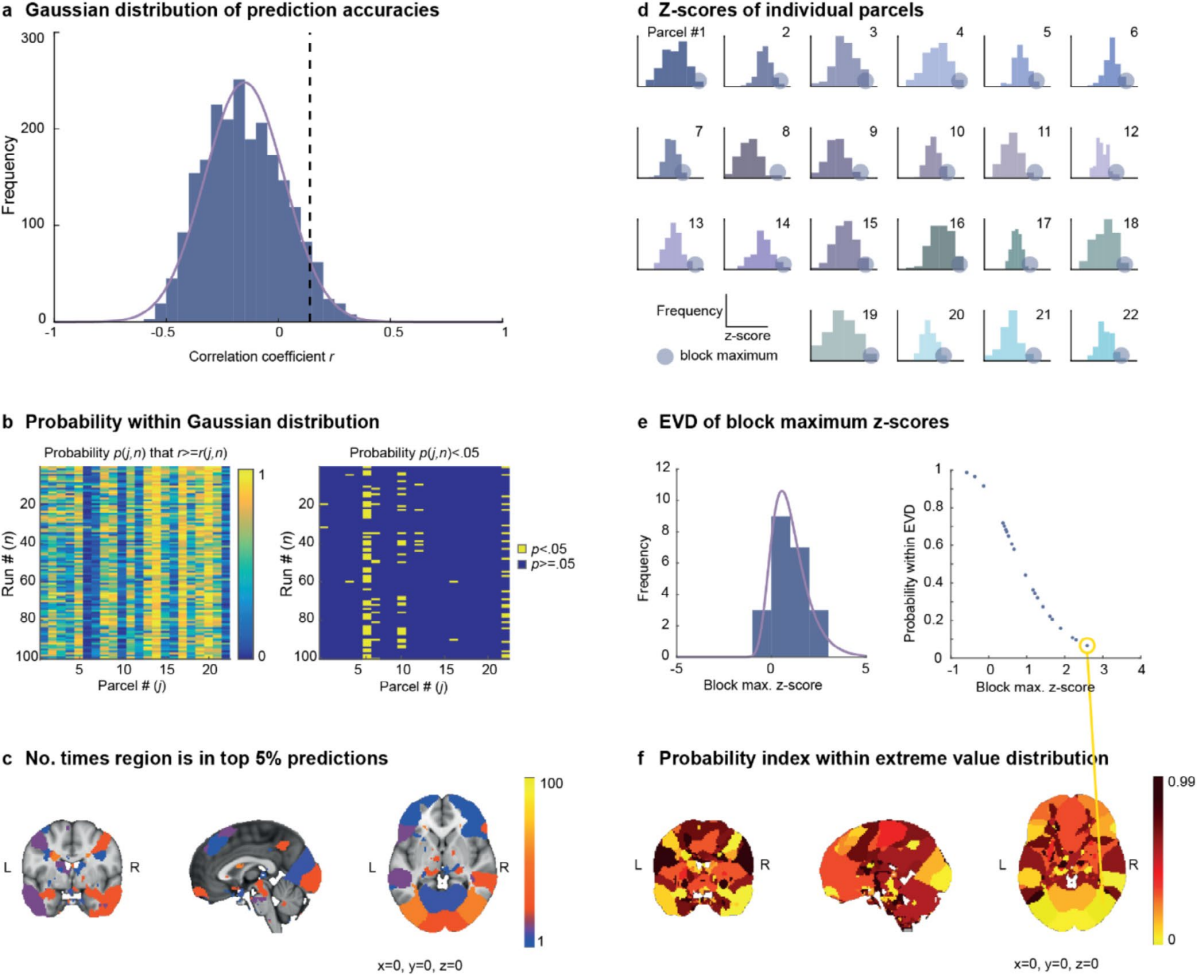
Across the whole brain, task activation is robustly better predicted in visual areas. (a) Histogram of prediction accuracies across all 22 parcels and 100 iterations and fitted Gaussian distribution. The dashed line represents the 95th percentile, that is, the top 5% of predictions. (b) Probability of obtaining prediction accuracy by parcel and run. (c) Regions that appear more than once in the top 5% predictions across the whole brain. (d) *z*‐scores and block maxima (the 10% best predicted runs) across runs for each parcel. (e) Histogram and fitted extreme value distribution (EVD) for 90% trimmed mean block maximum *z*‐scores from (d). (f) Probability index, that is, probability of obtaining the block max. *z*‐score given the PDF of the EVD from (e) across regions in the whole‐brain parcellation.

As for the local auditory cortex parcellation, we predicted interindividual differences in task activation for each parcel in the whole‐brain parcellation, and we ran each model 100 times. Figure 4a shows the histogram and fitted Gaussian distribution of the obtained prediction accuracies (correlation coefficients between predicted and true interindividual variability) across all parcels and runs, as well as the dashed line cut‐off for the 95th percentile. Plotting the probability of the obtained prediction accuracies by parcel and run (Figure 4b) shows that certain regions appear several times in the top 5% predictions, while other regions never appear. In the whole‐brain map, the region with the highest number of appearances in the 95th percentile is the bilateral visual association cortex (here Parcel #6) with 55 out of 100 runs.

We then considered, as for the local auditory cortex parcellation, the robust probabilistic index of the best predictions in each parcel. In the whole‐brain parcellation, the largest outlier (i.e., the parcel that is robustly best predicted) was again the bilateral visual association cortex (Parcel #6) with a block max. *z*‐score of 2.59 (*p* = 0.06).

## 4 Discussion

In this study, we aimed at predicting differences between individuals in brain activation patterns during melodic contour processing from their rsFC. We found that individual patterns of paradigm activation were significantly better predicted in some regions than in others. The best predicted parcels were sensory association cortices (visual and auditory), followed by primary sensory cortices (visual and motor), and areas belonging to the DMN (medial prefrontal cortex, posterior cingulate and hippocampus). In these regions, interindividual differences in paradigm activation between individuals could be accurately predicted, and the superiority of prediction accuracy in these regions was robust across randomised runs of the model.

One reason we expected individual differences to be more predictable in some parcels than in others is that not all regions are relevant for the paradigm at hand. The paradigm we employed specifically requires the processing of melodic contour, a higher‐level feature of auditory stimuli concerning the direction of a sequence of tones while disregarding the size of their pitch interval. In the local (auditory cortex) parcellation, we found that individual differences in paradigm activation could be best predicted in the right STG, a part of the auditory association cortices. This area is crucial for the processing of complex auditory representations, such as melodic contour: While more basic auditory features, such as pitch, are processed mainly in the primary auditory cortex, more abstract and complex melodic features are processed in areas further away from the primary auditory cortex, like the STG and parieto‐temporal junction (Stewart et al. 2006) (for a review, see Deouell et al. 2007). Thus, it appears that complex melodic processing is organised hierarchically, with higher‐level auditory regions involved in the recognition of melodic directionality (Patterson et al. 2002; Warren and Griffiths 2003). Brain areas involved in the processing of melodic contour have recently been investigated using multivariate pattern analysis (MVPA). These studies have found that regions in the right (Lee et al. 2011) or bilateral STG and superior temporal sulcus (Schindler et al. 2013) play a role in the processing of ascending or descending melodic contour by showing that local patterns of activity can accurately discriminate between contour categories. These findings are supported by lesion studies showing that lesions in the right superior temporal lobe impair the ability to discriminate the direction of pitch intervals, which forms the basis for melodic contour processing (Johnsrude et al. 2000). Additionally, only right‐lateralised lesions appear to cause deficits in melodic contour processing, while lesions in the left hemisphere do not affect this capacity (Peretz 1990). Behavioural work also supports the existence of a right‐hemisphere bias for the processing of melodic contour. In one experiment, McKinnon and Schellenberg (1997) presented listeners with a series of five tones in one ear and instructed them to make a forced‐choice judgement regarding the sequence's contour. Results indicated that performance was better when the sequence was presented to the left ear (i.e., first processed in the right hemisphere) as opposed to the right ear (left hemisphere). This provides evidence for the notion that the right hemisphere has an inclination to process melodic contour. The involvement of right STG/STS in the processing of contour features is also supported by neuroimaging work on language processing (Kyong et al. 2014). The right superior temporal regions exhibited the largest activity for spoken language with modulation of suprasegmental contour features. Our finding that the right STG can be better predicted than other parts of the auditory cortices may thus be due to this area's relevance to processing melodic contour, a central requirement of the paradigm. Interestingly, we did not observe higher predictability in Heschl's gyrus, despite its relevance for melodic contour processing. This indicates that task involvement alone does not explain the better predictability of a given brain region. While it is possible that a comparison between contour and interval processing could have informed this regional specificity, we here opted not to target interval deviants because we found no significant activation in previous experiments (Lumaca, Dietz, et al. 2021), which limits interpretability.

The second reason for differences between regions in predictability is that activation patterns in some brain regions may be more individual‐specific than in others. Several studies aiming to find individual differences in paradigm activation have found sensory (association) cortices to be best predicted, despite not being expected to be relevant to the paradigm at hand. Villalta‐Gil et al. (2017) investigated individual responses to fear paradigms, expecting to find correlations with activation in the amygdala, but found them reflected in the visual cortex instead. When averaging over 47 different paradigm contrasts, Lacosse et al. (2021) found individual differences in paradigm activation to be most predictable in the sensory (visual, auditory and motor) association cortices. Similarly, Mueller et al. (2013) found that individual variability in FC is higher in heteromodal association cortices compared to unimodal sensory cortices and that brain regions that have higher between‐participant variability in FC are better predictors of behaviour in several cognitive domains. Here, using an auditory paradigm with concurrent visual stimuli, we also found the visual association cortices to be best predicted in the global parcellation. Although these were not significant in the contrast between melodic contour deviants and standards in our previous task activation study, it is possible that visual processes, such as visual imagery or processing of the concurrent visual stimuli, differ between individuals. Within the local auditory cortex parcellation, the best predicted area is the right STG, which is considered to be an auditory association area (Zevin 2009). A recent study also investigated inter‐individual variability based on intrinsic rsFC and a passive listening paradigm in different sites within the auditory cortices in humans and macaques (Ren et al. 2021) and found the non‐primary auditory cortices to be the most distinct. In the brain, phylogenetically old regions like the brain stem and deep subcortical regions that serve fundamental survival functions may be more similar between individuals, while higher‐order areas in the neocortex may exhibit differences according to an individual's unique traits. Indeed, fibre connections in frontal and limbic areas differ little between individuals, while temporal and occipital regions are very diverse (Kerepesi et al. 2018). Inter‐individual variability of rsFC is larger in frontal and parietal cortices, phylogenetically and ontogenetically late‐developing regions (Kaas 2006; Smaers et al. 2011; Van Essen and Dierker 2007), whose protracted plasticity would expose them longer to experiential factors (Petanjek et al. 2011). Both paradigm relevance and a more general level of information about the individual that is intrinsic to sensory association areas may therefore influence predictability across brain regions.

While the prediction of individual differences in paradigm activation from rsFC has the potential to make an important link between fingerprinting studies and the understanding of cognitive function, it seems that more work is needed before this method can be reliably used across the brain in real‐life fMRI datasets. An evident limitation is the number of participants necessary for the estimation of these models. Although the sample in this study (*N* = 52) is large for an fMRI study, the optimal sample size may be more likely in the order of hundreds, such as in most of the studies previously reported (Cohen et al. 2020; Cole et al. 2016; Parker Jones et al. 2016; Tavor et al. 2016). This also means that, given our sample size, we cannot make claims about the extent to which our findings generalise to the general population. However, even using a sample size of *N* = 200, a recent replication study found that most methods that have been reported to be able to predict paradigm activation maps from rsFC are optimistic and in fact do not outperform simple baseline models (Lacosse et al. 2021). Improving these modelling efforts is an active area of research. It is possible, however, that the difficulty of predicting paradigm activation from rsFC lies in the nature of the data rather than in the details of the models. Additionally, it is possible that differences in signal quality across brain areas may affect predictability, since noisier voxels, for example, voxels more strongly affected by physiological or scanner artefacts, will be less predictable, while voxels with better signal‐to‐noise ratio or smaller variance will be more predictable.

While both structural connectivity and rsFC naturally vary greatly between participants, classic fMRI paradigms are designed to constrain brain activity to the studied function. Since the goal of these paradigms is to elucidate brain activity underlying a specific cognitive function by averaging across the group, they allow little variance between participants by definition. Large enough variance between participants is a fundamental requirement for a predictive model to perform well. Lacosse et al. (2021) also found in their study that only 1 of the 47 contrasts studied could reliably be predicted. It is possible that more naturalistic, unconstrained paradigms could be more easily predicted than the ones studied here and in previous studies.

Modelling individual paradigm activation patterns from rsFC is a worthy goal, since it may be of great clinical relevance for patients unable to complete a certain task. It has, for instance, been shown that it is possible to use predicted individual activity patterns to personalise surgical targets (Niu et al. 2021; Parker Jones et al. 2016).

In sum, we here show that differences between individuals in an auditory oddball paradigm of melodic contour can be best predicted in sensory association areas. Creating a sub‐parcellation of the auditory cortices made it possible to identify the right STG, an area that is both relevant to the paradigm and likely carries a high amount of individual information, as a site where differences between individuals in paradigm activation can be predicted well. The prediction of individual differences in a paradigm from rsFC depends on each brain area's relevance to the paradigm and its level of inter‐individual variability. While activation patterns in some areas may thus be similar across individuals, sensory association cortices are unique—akin to a fingerprint.

## Author Contributions

**Christine Ahrends:** conceptualisation, methodology, software, formal analysis, investigation, writing – original draft, visualisation. **Massimo Lumaca:** conceptualisation, data curation, writing – original draft, writing – review and editing. **Morten L. Kringelbach:** supervision, writing – review and editing. **Diego Vidaurre:** conceptualisation, methodology, software, writing – review and editing. **Peter Vuust:** resources, funding acquisition, supervision, writing – review and editing.

## Funding

This work was supported by the Carlsberg Foundation (CF23‐1716), the Novo Nordisk Foundation (NNF19OC‐0054895), the European Research Council (ERC‐StG‐2019‐850404) and the Danish National Research Foundation (DNRF117).

## Conflicts of Interest

The authors declare no conflicts of interest.

## Supporting information


**Figure S1:** Summary maps of group ICA parcellation of the whole brain (global).
**Figure S2:** Summary maps of group ICA parcellation of the auditory cortex (local).

## Data Availability

The data that support the findings of this study are available on request from the corresponding author. The data are not publicly available due to privacy or ethical restrictions.
