# Supplementary material for: Prediction of Individual Melodic Contour Processing in Sensory Association Cortices From Resting State Functional Connectivity
Source: Hum Brain Mapp. 2025 Nov 26;46(17):e70409. doi: 10.1002/hbm.70409 (PMC12648184; doi:10.1002/hbm.70409)
Supplement: Supplementary file 1 — Figure S1: Summary maps of group ICA parcellation of the whole brain (global). Figure S2: Summary maps of group ICA parcellation of the auditory cortex (local). [file HBM-46-e70409-s001.docx]

**Supplementary Material - Ahrends et al. Prediction of individual melodic contour processing in sensory association cortices from resting state functional connectivity**


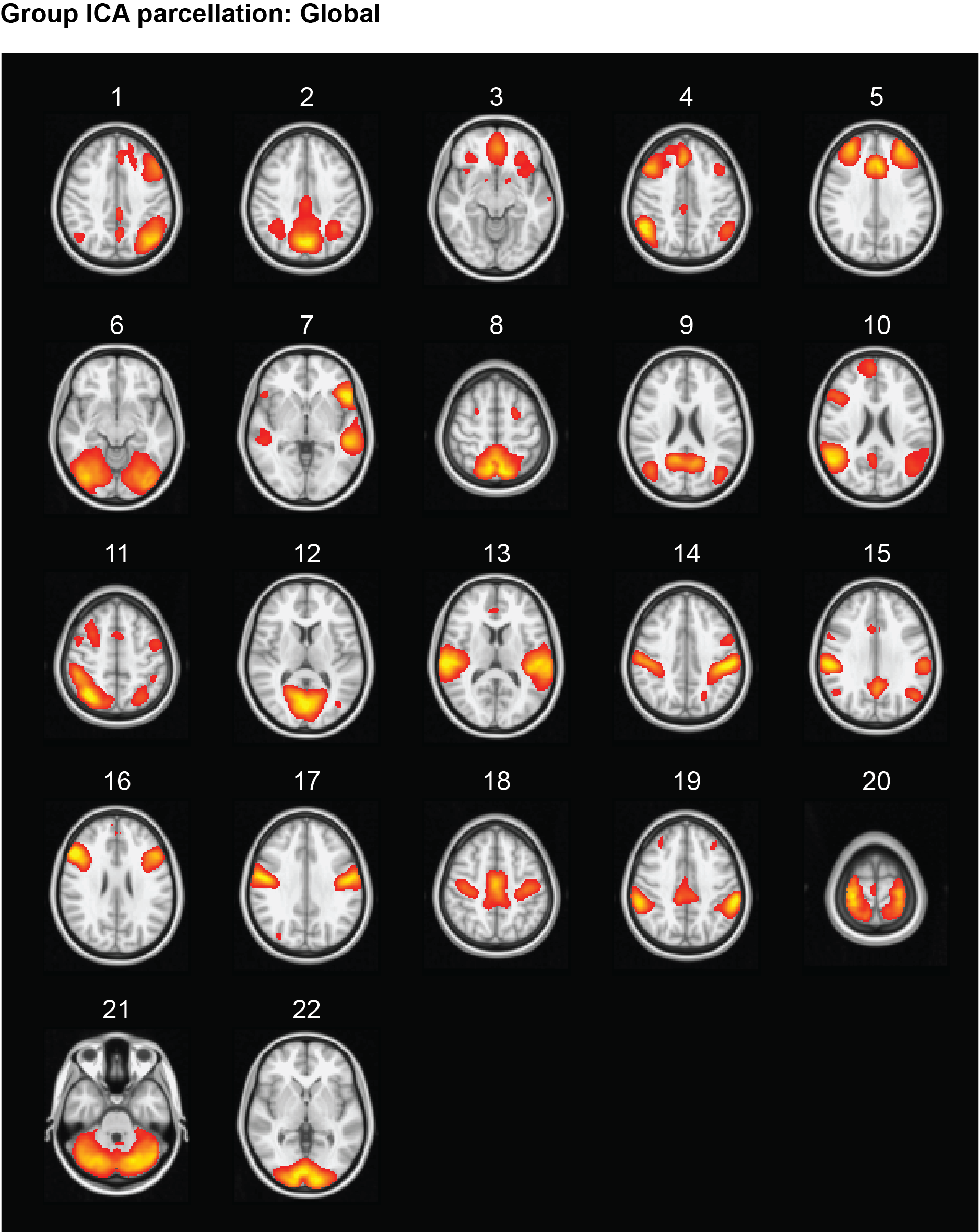


Figure 1 Summary maps of group ICA parcellation of the whole brain (global).


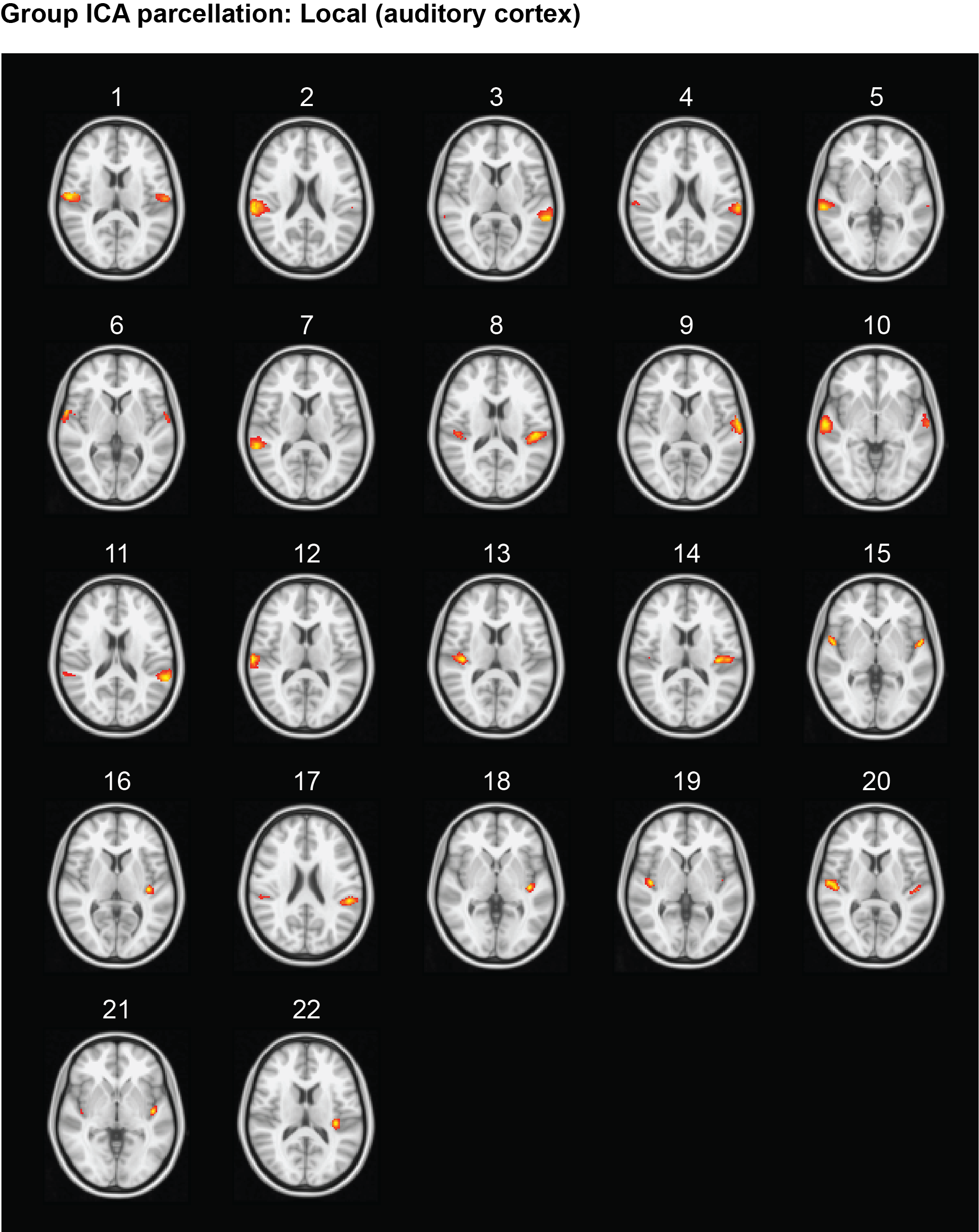


Figure 2 Summary maps of group ICA parcellation of the auditory cortex (local).
